# Aesculetin Accelerates Osteoblast Differentiation and Matrix-Vesicle-Mediated Mineralization

**DOI:** 10.3390/ijms222212391

**Published:** 2021-11-17

**Authors:** Woojin Na, Min-Kyung Kang, Sin-Hye Park, Dong Yeon Kim, Su Yeon Oh, Moon-Sik Oh, Sohyun Park, II-Jun Kang, Young-Hee Kang

**Affiliations:** Department of Food and Nutrition and Korean Institute of Nutrition, Hallym University, Chuncheon 24252, Korea; nsm0729@hanmail.net (W.N.); mitholy@hallym.ac.kr (M.-K.K.); shpark88@hallym.ac.kr (S.-H.P.); ehddus3290@naver.com (D.Y.K.); suy0411@naver.com (S.Y.O.); 2569227@naver.com (M.-S.O.); sopark@hallym.ac.kr (S.P.)

**Keywords:** aesculetin, collagen mineralization, hydroxyapatite, matrix vesicles, non-collagenous proteins, osteoblast differentiation

## Abstract

The imbalance between bone resorption and bone formation in favor of resorption results in bone loss and deterioration of bone architecture. Osteoblast differentiation is a sequential event accompanying biogenesis of matrix vesicles and mineralization of collagen matrix with hydroxyapatite crystals. Considerable efforts have been made in developing naturally-occurring plant compounds, preventing bone pathologies, or enhancing bone regeneration. Coumarin aesculetin inhibits osteoporosis through hampering the ruffled border formation of mature osteoclasts. However, little is known regarding the effects of aesculetin on the impairment of matrix vesicle biogenesis. MC3T3-E1 cells were cultured in differentiation media with 1–10 μM aesculetin for up to 21 days. Aesculetin boosted the bone morphogenetic protein-2 expression, and alkaline phosphatase activation of differentiating MC3T3-E1 cells. The presence of aesculetin strengthened the expression of collagen type 1 and osteoprotegerin and transcription of Runt-related transcription factor 2 in differentiating osteoblasts for 9 days. When ≥1–5 μM aesculetin was added to differentiating cells for 15–18 days, the induction of non-collagenous proteins of bone sialoprotein II, osteopontin, osteocalcin, and osteonectin was markedly enhanced, facilitating the formation of hydroxyapatite crystals and mineralized collagen matrix. The induction of annexin V and PHOSPHO 1 was further augmented in ≥5 μM aesculetin-treated differentiating osteoblasts for 21 days. In addition, the levels of tissue-nonspecific alkaline phosphatase and collagen type 1 were further enhanced within the extracellular space and on matrix vesicles of mature osteoblasts treated with aesculetin, indicating matrix vesicle-mediated bone mineralization. Finally, aesculetin markedly accelerated the production of thrombospondin-1 and tenascin C in mature osteoblasts, leading to their adhesion to preformed collagen matrix. Therefore, aesculetin enhanced osteoblast differentiation, and matrix vesicle biogenesis and mineralization. These findings suggest that aesculetin may be a potential osteo-inductive agent preventing bone pathologies or enhancing bone regeneration.

## 1. Introduction

The ossification process is tightly regulated by specialized osteoblasts, but its dysregulation results in aberrant mineralization of bones or ectopic calcification such as osteogenesis imperfecta and osteoporosis [1,2]. Osteoblasts differentiate from bone marrow mesenchymal progenitors via the distinct process involving cell proliferation, extracellular matrix formation and maturation, and bone matrix mineralization [1,3]. Subsequently, osteoblasts further differentiate into stellate cells (i.e., osteocytes), which populate interconnected canaliculi within the bone matrix [4]. Osteoblasts regulate bone matrix formation and mineralization directly by their own synthetic activities, and indirectly by paracrine effects of bone resorption on osteoclasts [5]. Osteoblast differentiation known as osteoblastogenesis, is regulated by a variety of growth factors and cytokines including bone morphogenetic protein-2 (BMP-2), osteocalcin, and osteopontin in a network manner [6,7]. The BMP-2 signaling induces pluripotent stem cell differentiation into bone cells via multiple signal pathways that promote osteogenesis [8]. In addition, osteoblast differentiation is orchestrated by Wnt signaling pathway and the network of transcription factors such as Runt-related transcription factor (Runx) 1/2 and osterix [7,9]. Although the differentiating osteoblast lineage, pre-osteoblasts, retain a proliferative capacity, they express several proteins associated with the mature osteoblast phenotype, including alkaline phosphatase (ALP) and osteopontin [6]. During early proliferation of osteoblasts increased collagen type 1 enhances ALP expression, leading to bone matrix maturation and mineralization [7].

The mature osteoblasts lie adjacent to newly synthesized osteoid and produce the bone mineral hydroxyapatite that is deposited into the organic matrix, forming a dense mineralized matrix [9,10]. Hydroxyapatite crystals present in bone is interspersed in a collagen matrix in a highly regulated manner [11,12]. During bone mineralization of mature osteoblasts, the organic osteoid matrix becomes filled with calcium phosphate nanocrystals in a specific and well-organized way [13,14]. In addition, the matrix is primarily composed of collagen type 1 fibrils arranged by axial and radial aggregation in a specific tertiary structure [15,16]. Calcium phosphate crystals (Ca^2+^/PO_4_^3−^) grow out of matrix vesicles through rupture of their membrane to form calcifying nodules [12]. Small extracellular matrix vesicles and proteins secreted by mature osteoblasts are observed in the pre-mineralized matrix of bone surfaces, inducing the nucleation and subsequent growth of calcium phosphate crystals inside [12,17]. Accumulation of calcium phosphate inside the matrix vesicles initiates crystalline nucleation associated with the inner leaflet of the matrix vesicles. However, the molecular mechanisms of the biogenesis of matrix vesicles and processes leading to mineral/apatite formation are still unclear. Several enzymes and transporters such as ecto-nucleotide pyrophosphatase/phosphodiesterase 1, PHOSPHO1, and tissue-nonspecific alkaline phosphatase (TNSALP) on matrix vesicle membranes are involved in the growth and burst of calcium phosphate crystals [18].

**Figure 1:**
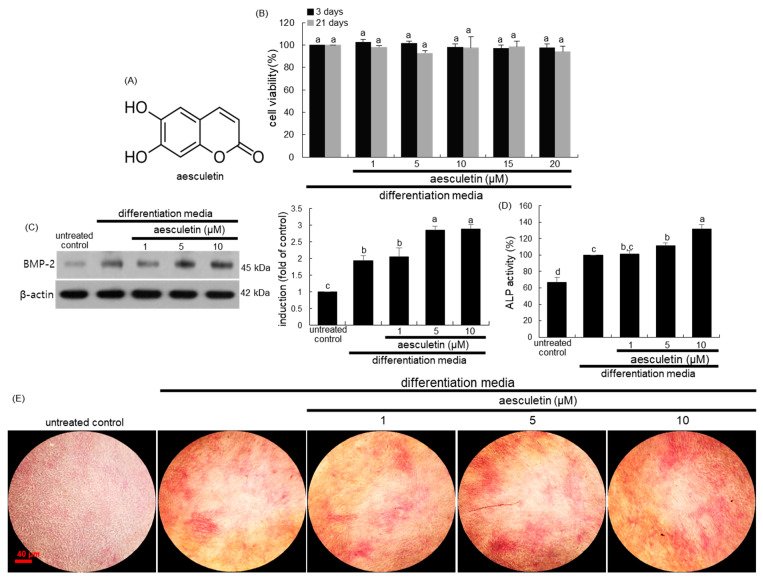
Chemical structure of aesculetin (**A**), cytotoxicity of MC3T3-E1 cells by 1–20 μM aesculetin (**B**), upregulation of bone morphogenetic protein-2 (BMP-2) expression (**C**) and alkaline phosphatase (ALP) activity (**D**) and staining (**E**) by aesculetin. MC3T3-E1 cells were cultured for 3 days and 21 days with 1–20 µM aesculetin in differentiation media. Cell viability was measured by MTT assay (**B**). Bar graphs for viability (mean ± SEM, n = 3) was expressed as percent cell survival compared to untreated cells. Further, MC3T3-E1 cells were cultured in differentiation media in the absence or presence of 1–10 μM aesculetin for three days (BMP-2) and seven days (ALP). Whole cell lysates were subject to SDS-PAGE and Western blot with a specific antibody against BMP-2 (**C**). β-Actin was used as an internal control. The bar graphs (mean ± SEM, n = 3) represent quantitative results of blots obtained from a densitometer. The ALP activity (**D**, mean ± SEM, n = 6) was measured at λ = 405 nm. The ALP staining was visualized under light microscopy (**E**, 4 separate experiments). Scale bar = 40 μm. Respective values not sharing a small letter are different at *p* < 0.05.

The commitment, differentiation, and mineralization of osteoblasts have been applied towards the development of new therapeutic alternatives for bone diseases. Inflammatory factors enhance the osteogenic capacity of mesenchymal stem cells after lineage commitment [19]. Recently, novel epigenetic regulators open a new window for targeting osteoblast differentiation [20]. On the other hand, considerable efforts have been made in developing natural plant-derived compounds for improving the treatment of bone-decreasing diseases and enhancing bone regeneration [21,22]. The isoflavone calycosin-7-O-β-d-glucopyranoside stimulates osteoblast differentiation through regulating the BMP/Wnt signaling [22]. Our previous study showed that the dihydrochalcone phlorizin stimulated osteoblastogenic bone formation through enhancing β-catenin activity via glycogen synthase kinase-3β (GSK-3β) inhibition in a model of senile osteoporosis [23]. However, the mechanistic efficacy of these compounds in bone mineralization remains elusive. The role of matrix vesicles in bone formation and mineralization could help to target bone pathologies or regeneration. In our recent study, naturally-occurring aesculetin attenuated osteoclast differentiation and impaired formation of the putative ruffled border of mature osteoclasts [24]. However, little is known regarding the effects of aesculetin on the matrix vesicle secretion. Based on the evidence that osteoblastogenesis relies on molecular apparatus linked to the biogenesis of osteo-inductive matrix vesicles and processes leading to bone mineral hydroxyapatite formation [25], the present study examined whether aesculetin (Figure 1A) enhanced osteogenesis through stimulating bone-targeting matrix vesicle secretion and collagen mineralization of mature osteoblasts.

## 2. Results

### 2.1. Initiation of Osteoblastic Differentiation by Aesculetin

This study investigated whether submicromolar aesculetin stimulated the initiation of osteoblast differentiation. It should be noted that there was no significant toxicity of aescueltin observed in MC3T3-E1 cells incubated in differentiation media for 3 days or 21 days (Figure 1B). The BMP-2 induces the differentiation of stem cells into bone cells through activation of multiple signal pathways [8]. When MC3T3-E1 cells were cultured for 3 days in differentiation media containing 50 μg/mL ascorbic acid and 100 nM dexamethasone, the BMP-2 expression was highly elevated (Figure 1C). Such elevation was further enhanced in differentiation media with ≥5 μM aesculetin.

ALP is expressed during osteoblast differentiation as an early marker gene and is observed on the cell surface and in matrix vesicles [26,27]. The ALP activity was elevated in MC3T3-E1 cells cultured in differentiation media for seven days (Figure 1D). When non-toxic aesculetin at 1–10 μM was treated to MC3T3-E1 cells cultured in differentiation media, The ALP activity was further enhanced in a dose-dependent manner. Consistently, the differentiated MC3T3-E1 cells showed strong ALP staining, which was furthermore boosted in the presence of aesculetin (Figure 1E). During osteoblast proliferation, the enhanced ALP expression facilitates bone matrix maturation and mineralization [7].

### 2.2. Induction of Mid-to-Late Stage of Osteoblastic Differentiation by Aesculetin

**Figure 2:**
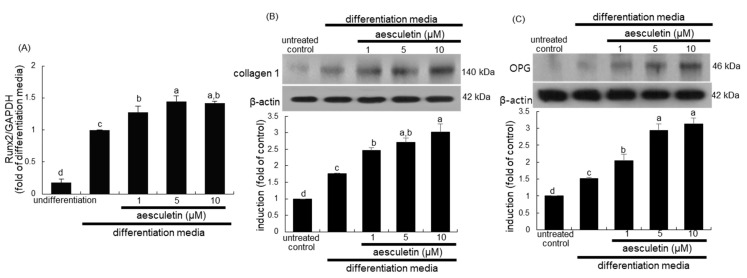
Upregulation of Runt-related transcription factor 2 (Runx2) transcription (**A**), and expression of collagen type 1 (**B**) and osteoprotegerin (OPG, **C**) by aesculetin. MC3T3-E1 cells were cultured for nine days with 1–10 µM aesculetin in differentiation media. The transcription of Runx2 was measured by real-time polymerase chain reaction assay, and glyceraldehyde 3-phosphate dehydrogenase (GAPDH) gene was used for the internal controls (**A**). For the expression of collagen type 1 and OPG, whole cell lysates were subject to SDS-PAGE and Western blot with a specific antibody against collagen type 1 or OPG (**B**,**C**). β-Actin was used as an internal control. The bar graphs represent quantitative results of blots obtained from a densitometer. Respective values (mean ± SEM, n = 3) not sharing a small letter are different at *p* < 0.05.

This study examined whether aesculetin augmented the mid-to-late differentiation stage of osteoblastic differentiation during the 21 day-osteoblastic differentiation. The transcriptional expression of Runx2 that is essential for osteoblast differentiation increases in immature osteoblasts and declines in mature osteoblasts [2]. Consistent with our previous findings [23], the transcription of Runx2 was highly enhanced in MC3T3-E1 cells cultured for nine days in differentiation media (Figure 2A). The treatment of aesculetin to differentiating osteoblast lineage further elevated such transcriptional expression. In addition, the protein expression of collagen type 1 and osteoprotegerin (OPG) was much more enhanced by adding ≥1 μM aesculetin to MC3T3-E1 cells cultured for nine days in differentiation media (Figure 2B,C). Thus, aesculetin may accelerate the mid-to-late stage of osteoblastic differentiation.

### 2.3. Upregulation of Later Stages of Osteoblastic Differentiation by Aesculetin

**Figure 3:**
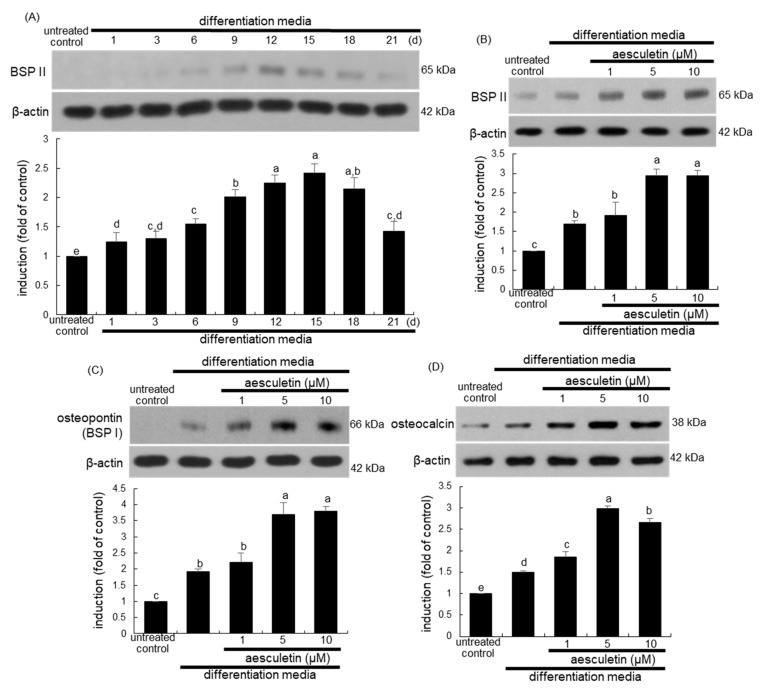
Temporal induction of bone sialoprotein (BSP) II (**A**) and elevation of induction of BSP II (**B**), osteopontin (**C**), and osteocalcin (**D**) by aesculetin in osteoblastic MC3T3-E1 cells. MC3T3-E1 cells were cultured in differentiation media in the absence or presence of 1–10 μM aesculetin for up to 21 days. For the measurement of expression of BSP II, osteopontin and osteocalcin of aesculetin, MC3T3-E1 cells were differentiated for 15 days. Whole cell lysates were subject to SDS-PAGE and western blot with a specific antibody against BSP II, osteopontin or osteocalcin. β-Actin was used as an internal control. The bar graphs (mean ± SEM, n = 3) represent quantitative results of blots obtained from a densitometer. Respective values not sharing a small letter are different at *p* < 0.05.

The non-collagenous bone sialoprotein (BSP) II and osteopontin also known as BSP I are necessary for the initiation of bone mineralization [28]. This study investigated the time course-induction of BSP II in differentiating MC3T3-E1 cells. The expression of BSP II was induced in a temporal manner, with its peak levels at 12–15 days after osteogenic differentiation (Figure 3A). The BSP II induction was further promoted by treating ≥5 μM aesculetin to 15 day-differentiating MC3T3-E1 cells (Figure 3B). Additionally, the osteopontin expression of osteoblastic MC3T3-E1 cells was markedly enhanced by ≥5 μM aesculetin (Figure 3C). Our previous study revealed that the osteocalcin transcription was temporally enhanced to maximum on the day 15 after osteogenic differentiation [23]. The osteocalcin expression of osteoblastic MC3T3-E1 cells was markedly enhanced by ≥1 μM aesculetin (Figure 3D). Accordingly, both non-collagenous proteins of BSP II, osteopontin, and osteocalcin were induced by aesculetin ahead of the mineralization front as mineral crystal nucleators.

### 2.4. Increased Terminal Differentiation Leading to Mineralization by Aesculetin

**Figure 4:**
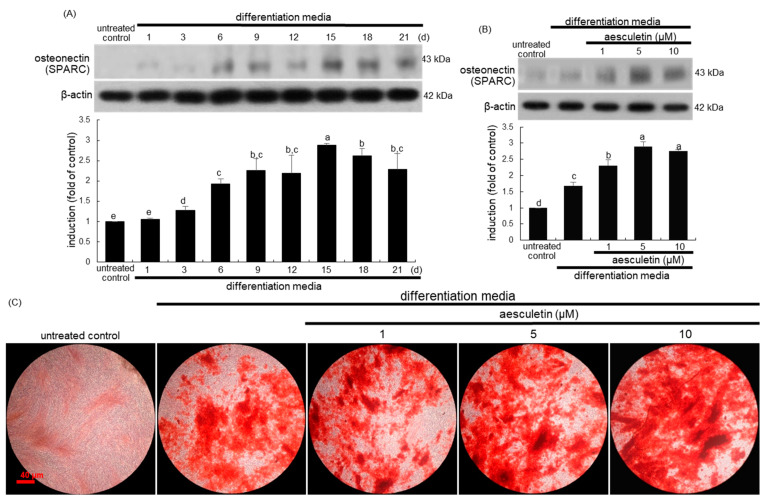
Temporal induction of osteonectin (**A**), induction elevation of osteonectin (**B**) and formation of calcium nodules (**C**) by aesculetin in osteoblastic MC3T3-E1 cells. MC3T3-E1 cells were cultured in differentiation media in the absence or presence of 1–10 μM aesculetin for up to 21 days. To examine the osteonectin expression of aesculetin, MC3T3-E1 cells were differentiated for 15 days. Whole cell lysates were subject to SDS-PAGE and Western blot with a specific antibody against osteonectin. β-Actin was used as an internal control. The bar graphs (mean ± SEM, n = 3) represent quantitative results of blots obtained from a densitometer. Respective values not sharing a small letter are different at *p* < 0.05. Matrix mineralization was measured by Alizarin red S staining (**C**). Microphotographs were representative of 21 day-grown osteoblasts on the wells. Heavy reddish staining of Alizarin red S is proportional to the area of mineralized matrix in osteoblastic MC3T3-E1 cells. Scale bar = 40 μm. The calcium nodules were visualized under light microscopy (5 separate experiments).

Osteonectin is responsible for the formation of hydroxyapatite crystals and mineralized matrix [28]. The induction of osteonectin also known as SPARC reached the peak levels at 15–18 days after osteogenic differentiation in a time course-dependent manner (Figure 4A). The osteonectin expression was further induced by adding ≥1 μM aesculetin to 18 day-differentiating MC3T3-E1 cells (Figure 4B).

The formation of calcium nodules is one of characteristics of mature osteoblasts [29]. Alizarin red S staining showed that no noticeable calcium deposit was observed in undifferentiated MC3T3-E1 cells (Figure 4C). However, there was strong reddish staining in MC3T3-E1 cells differentiated for 21 days. Furthermore, 1–10 μM aesculetin stimulated the calcium deposition, indicating that aesculetin increased the calcified matrix production (Figure 4C). Accordingly, it can be assumed that the completion of osteoblastic differentiation is associated with matrix synthesis and mineralization.

### 2.5. Incorporation of Osteoblasts into the Matrix by Aesculetin

**Figure 5:**
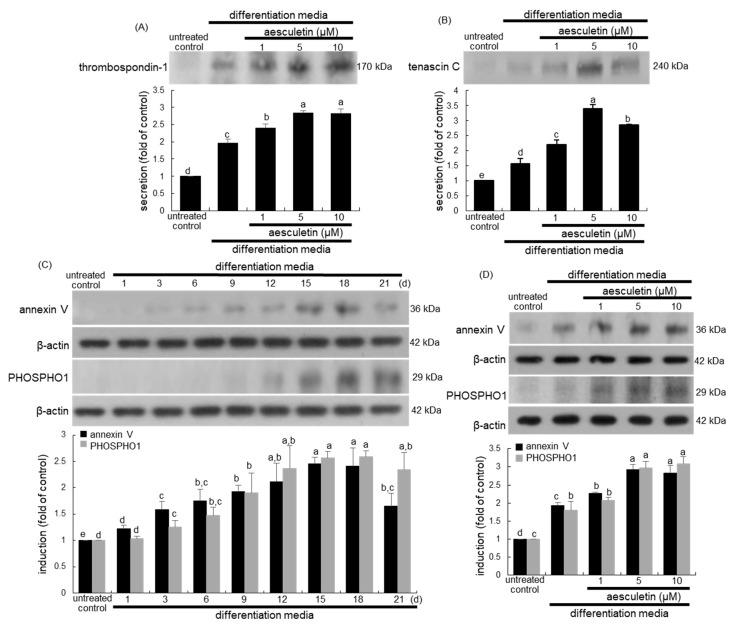
Induction of thrombospondin-1 and tenascin C by aesculetin (**A**,**B**), temporal induction of annexin V and PHOSPHO 1 (**C**), and elevation of annexin V and PHOSPHO 1 with aesculetin (**D**). MC3T3-E1 cells were cultured in differentiation media in the absence or presence of 1–10 μM aesculetin up to 21 days. To measure the effects of aesculetin on expression of annexin V, PHOSPHO 1, thrombospondin-1, and tenascin C, cells were differentiated for 18 days (**A**,**B**,**D**). Whole cell lysates were subject to SDS-PAGE and Western blot with a specific antibody against annexin V, PHOSPHO 1, thrombospondin-1 or tenascin C. β-Actin was used as an internal control. The bar graphs (mean ± SEM, n = 3) represent quantitative results of blots obtained from a densitometer. Respective values in bar graphs not sharing a small letter are significantly different at *p* < 0.05.

Thrombospondin-1 and collagen 1 are colocalized in intracellular vesicles and on extracellular collagen fibrils [30]. This study investigated that aesculetin boosted the thrombospondin-1 secretion from differentiating MC3T3-E1 cells. In ≥1 μM aesculetin-treated differentiating MC3T3-E1 cells, the thrombospondin-1 secretion was markedly accelerated, suggesting the increased formation of intracellular vesicles (Figure 5A). On the other hand, this study investigated that aesculetin promoted the tenascin C secretion from differentiating MC3T3-E1 cells for 15–18 days. Tenascins regulate cell interaction with the surrounding pericellular matrix and influence osteoblast adhesion and differentiation within bone [31]. The treatment of submicromolar aesculetin to differentiating osteoblasts further stimulated the tenascin C production (Figure 5B), leading to tight adhesion of mature osteoblasts to the bone matrix.

### 2.6. Formation of Matrix Vesicles for Bone Mineralization by Aesculetin

This study investigated that aesculetin stimulated matrix vesicle-mediated mineralization in differentiating osteoblasts. Evidence shows that osteoblasts and chondrocytes shed a subset of extracellular bilayer vesicles known as matrix vesicles that contain phosphatases, calcium, and inorganic phosphate [12,17,25,32]. In addition, the calcium-binding annexin V and inorganic phosphate-generating PHOSPHO1are concentrated in or near the membrane of matrix vesicles present in mineralizing regions of bone and growth plate, and are involved in initiating the formation of mineral crystals within matrix vesicles [33,34]. The annexin V expression was temporally induced on the matrix vesicle membrane during the 21 day-differentiation of MC3T3-E1 cells (Figure 5C). Additionally, the PHOSPHO1 expression was increased maximally at 18–21 days after osteoblastic differentiation (Figure 5D). These results indicate that annexin V and PHOSPHO1 play a role in the initiation of matrix mineralization. When ≥5 μM aesculetin was treated to differentiating MC3T3-E1 cells, the expression of annexin V and PHOSPHO1 was highly enhanced (Figure 5D). Thus, aesculetin may promote matrix vesicle-mediated bone mineralization.

### 2.7. Induction of Collagen Mineralization by Aesculetin

**Figure 6:**
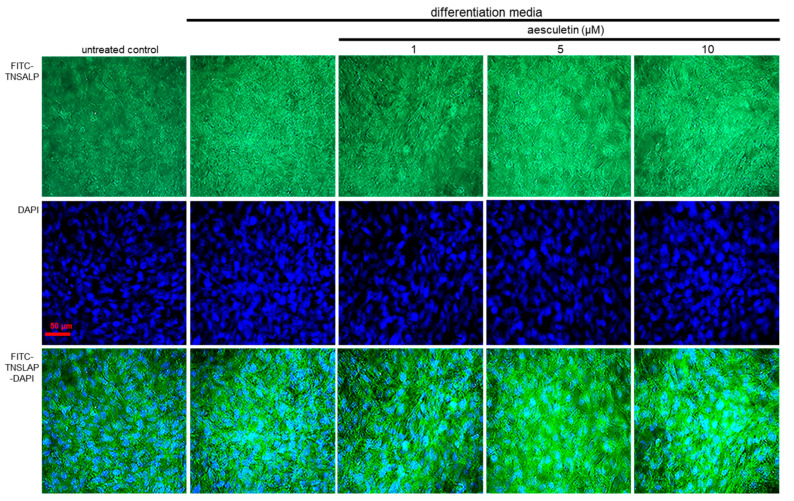
Immunofluorocytochemical analysis showing formation of tissue-nonspecific alkaline phosphatase (TNSALP) by aesculetin. MC3T3-E1 cells were cultured in differentiation media in the absence or presence of 1–10 μM aesculetin for 21 days. The matrix vesicle formation was confirmed by FITC-green staining of TNSALP on the top of differentiated MC3T3-E1 cells (n = 3). Nuclear staining of these cells was carried out with 4′, 6-diamidino-2-phenylindole (DAPI, blue), and visualized under light microscopy. Scale bar = 50 μm.

Although calcium ions are abundant in the tissue fluid close to the matrix vesicles, phosphate ions are entered into matrix vesicles through the mediation of several enzymes and transporters including ecto-nucleotide pyrophosphatase/phosphodiesterase 1 and TNSALP [11,12]. In order to detect TNSALP on matrix vesicles, this study conducted with green FITC-conjugated anti-TNSALP. There was strong staining observed on the top of differentiated osteoblasts for 21 days, indicating that the TNSALP on matrix vesicles was induced (Figure 6). When ≥5 µM aesculetin was administered to differentiated MC3T3-E1 cells, the green staining of TNSALP was further enhanced in the extracellular space and on matrix vesicles. Thus, aesculetin may increase the formation of matrix vesicles, leading to bone matrix formation and mineralization.

**Figure 7:**
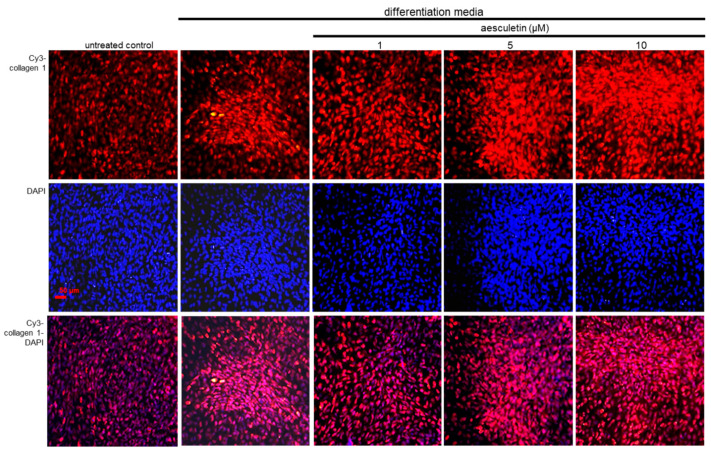
Immunofluorocytochemical analysis showing production of collagen type 1 by aesculetin. MC3T3-E1 cells were cultured in differentiation media in the absence or presence of 1–10 μM aesculetin for 21 days. The collagen type 1 production was confirmed by Cy3-red staining of collagen 1 on the top of differentiated MC3T3-E1 cells (n = 3). Nuclear staining of these cells was carried out with 4′, 6-diamidino-2-phenylindole (DAPI, blue). Scale bar = 50 μm.

Collagen type 1, most of the entire collagen content of bone, plays a role in controlling bone strength [35]. Immunocytochemical staining revealed that red Cy3-conjugated collagen 1 was highly induced in differentiated MC3T3-E1 cells for 21 days (Figure 7). When ≥5 µM aesculetin was treated to 1, the red Cy3-conjugated collagen 1 was elevated inside cells and in extracellular matrix (Figure 7). Accordingly, aesculetin may promote the formation of collagenous bone matrix.

## 3. Discussion

**Figure 8:**
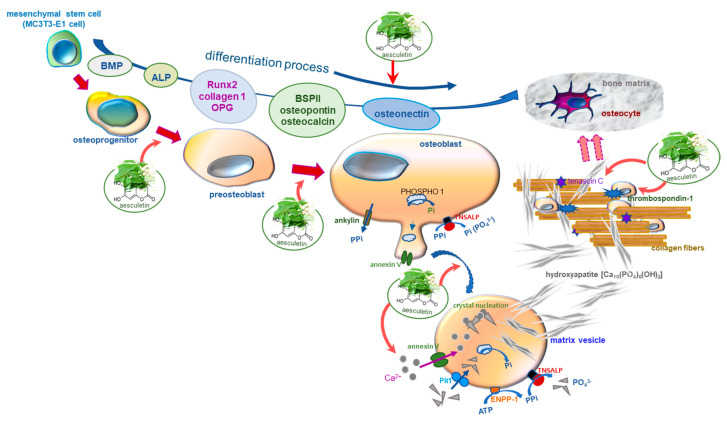
Schematic diagram showing the effects of aesculetin on osteoblastogenesis and collagen mineralization. The arrows indicate activation by aesculetin. ALP, alkaline phosphatase; BMP-2, bone morphogenetic protein-2; BSP, bone sialoprotein; OPG, osteoprotegerin; Runx2, Runt-related transcription factor 2; TNSALP, tissue-nonspecific alkaline phosphatase.

Nine main findings were extracted from this study. (1) Submicromolar aesculetin further enhanced the BMP-2 expression of MC3T3-E1 cells differentiated for three days, triggering the initiation of osteoblastic differentiation. (2) The presence of aesculetin dose-dependently boosted the ALP activation in MC3T3-E1 cells differentiated for seven days. (3) The expression of collagen type 1 and OPG was strengthened by ≥1 μM aesculetin in MC3T3-E1 cells differentiated for nine days along with the enhanced transcription of Runx2, indicating that aesculetin may accelerate the mid-to-later stage of osteoblast differentiation. (4) When ≥1–5 μM aesculetin was added to differentiating MC3T3-E1 cells for 15 days, the induction of BSP II, osteopontin, and osteocalcin was markedly enhanced, leading to the mineralization front as mineral crystal nucleators. (5) Osteonectin was further induced by supplying ≥1 μM aesculetin to differentiating MC3T3-E1 cells for 18 days, facilitating formation of hydroxyapatite crystals and mineralized matrix. (6) Aesculetin stimulated the calcium nodule formation and mineralization in differentiating MC3T3-E1 cells for 21 days. (7) The expression of annexin V and PHOSPHO 1 was further augmented in ≥5 μM aesculetin-treated differentiating MC3T3-E1 cells, initiating formation of mineral crystals within matrix vesicles leading to matrix vesicle-mediated bone mineralization. (8) The secretion of thrombospondin-1 and tenascin C was markedly accelerated in differentiating MC3T3-E1 cells for 18 days. (9) When ≥5 µM aesculetin was administered to differentiating MC3T3-E1 cells, the levels of TNSALP and collagen type 1 within the extracellular space and on matrix vesicles were further enhanced. Accordingly, aesculetin accelerated osteoblast differentiation, matrix vesicle biogenesis and mineralization, and enhanced adhesion of mature osteoblasts to collagenous bone matrix (Figure 8). These findings suggest that aesculetin may be a potential osteo-inductive agent preventing bone-degrading pathologies or enhancing bone regeneration.

Osteoblasts are differentiated from mesenchymal progenitors via the well-defined sequential process entailing cell proliferation, extracellular matrix formation and maturation, and bone matrix mineralization [1,3]. Osteoblasts further differentiate into osteocytes, which are embedded in calcified bone matrix [1,4]. Much knowledge has been gained in relation with the multiple factors and signaling networks regulating osteoblast differentiation at a molecular level [9,36]. Osteoblast differentiation is controlled in a network manner by a variety of factors such as BMP-2, Runx2, Osterix, transforming growth factor-β, and Hedgehog [7,9,36]. BMP regulates the development of bone and cartilage by interacting with several transcription factors [37]. In addition, the BMP-2 signaling induces pluripotent stem cell differentiation into bone cells via multiple signal pathways that promote osteogenesis [8]. Especially, the Runx2 regulation network is critical for osteoblast differentiation, which requires BMP signaling for the induction of osteoblast gene expression [37,38]. As expected, the current study showed that the BMP-2 expression was enhanced at the early stage of osteoblastic differentiation of MC3T3-E1 cells. In addition, the increased BMP-2 signaling accompanied ALP activation and Runx2 transcription as well as induction of collagen type 1 and OPG, all being responsible for the early to middle stages of osteoblast differentiation. In fact, increased collagen 1 expression and ALP activation can facilitate bone matrix maturation and mineralization at late stages of differentiation [7].

The osteogenic differentiation is accomplished by the sequential expression of non-collagenous matrix proteins of osteopontin, BSP II, osteocalcin and osteonectin at different stages of osteoblastic differentiation, in association with extracellular matrix mineralization [6,7,28]. This study found that the non-collagenous BSP II, osteopontin, osteocalcin and osteonectin were involved in operating the mid-to-late stages of osteoblastic differentiation. One study shows that osteocalcin is expressed during bone nodule formation and bone matrix mineralization [3]. Osteocalcin, osteopontin, and BSP II bind to the bone mineral via mineral-binding Gla groups or acidic groups and is adsorbed to bone [39]. In addition, collagen 1, osteonectin and small proteoglycans are incorporated into collagen fibrils present in the bone matrix [39,40]. On the other hand, pathological anomalies of the molecular crosstalk between collagen and non-collagenous matrix proteins can cause malformations and defects including osteoporosis [40]. This study revealed that mature osteoblasts differentiated from MC3T3-E1 cells for 21 days fully formed bone nodules, indicative of bone matrix mineralization. One can assume that collagen and key non-collagenous matrix proteins may be properly interconnected during bone matrix formation. Targeted osteoblast differentiation entailing sequential induction of non-collagenous matrix proteins can be applied towards the development of regenerative strategies in bone remodeling and new therapeutic alternatives for human bone diseases.

Potential anti-osteoporotic agents from plants have been investigated for their pathophysiological and pharmacological properties [41]. Considerable attempts have been made to highlight natural plant-derived compounds with possible anti-osteoporosis properties [21,22]. Our previous investigation found that fisetin and phloretin inhibited osteoclastic differentiation and bone resorption [42,43]. In addition, phlorizin promoted β-catenin-dependent osteoblastogenic bone formation via GSK-3β inhibition in a model of senile osteoporosis [23]. The milk thistle flavonoid silymarin shows osteogenic activity in osteoblasts and tibia-fractured mice [44]. Other investigation shows that calycosin glucopyranoside stimulates osteoblastic differentiation via regulation of BMP/Wnt signaling [22]. However, the mechanistic actions of these compounds in bone matrix formation and mineralization remain elusive. Our recent study found that aesculetin attenuated osteoclastogenesis through inhibition of the formation of putative ruffled border within mature osteoclasts [24]. This study further revealed that aesculetin accelerated osteoblastic differentiation and bone collagenous matrix formation. However, little is known regarding the effects of aesculetin on the biogenesis of matrix vesicles and leading to mineral hydroxyapatite formation. The contribution of aesculetin to the formation of matrix vesicles could help to antagonize bone pathologies or improve bone regeneration.

Matrix vesicles play a role in matrix mineralization, which can feature bone-targeting and osteo-inductive properties [25]. Biogenesis of polarized matrix vesicles occurs in selected areas of developing organic matrix of differentiating growth plate chondrocytes, osteoblasts, and odontoblasts [9,25,32]. The apatitic bone mineral crystals are formed within matrix vesicles of mature osteoblasts located at sites of initial calcification in cartilage, bone, and predentin with the assistance of phosphatases and calcium-binding molecules [10,18,32]. Subsequently, preformed hydroxyapatite crystals are exposed to the extracellular space across the matrix vesicle membrane, leading to biomineralization. This study found that calcium-binding annexin V, TNSALP and inorganic phosphate-generating PHOSPHO 1 were induced in the later stages of osteogenic differentiation, indicating that mineral crystal formation and matrix vesicle-mediated bone mineralization occurred in fully-matured osteoblasts. Consistently, Alizarin red S-stained calcium nodules were observed in mature osteoblasts differentiated for 21 days. In addition, the immunofluorescence staining showed that TNSALP and collagen type 1 were enhanced in mineralized extracellular matrix space. Furthermore, the increased production of thrombospondin-1 and tenascin C indicated that mature osteoblasts were entrapped in the mineralized collagen fibrils within bone matrix. Based on the evidence that osteoblastic osteogenesis depends on the biogenesis of matrix vesicles [25], potential therapeutic use of matrix vesicle mimetics such as proteoliposomes and polymeric vesicles is a challenge initiating skeletal mineralization [25,32].

Although molecular mechanisms underlying the biogenesis and function of matrix vesicles are still unclear, novel approaches stimulating secretion of matrix vesicles and use of biomimetic models could be potential strategies targeting against bone mineralization disorders. It has been shown that the introduction of calcium and phosphate triggers the secretion of matrix vesicles of osteoblasts [25,45]. Several studies report that administration of osteoblast-derived extracellular vesicles is osteo-inductive for bone mineralization disorders such as osteoporosis or fracture healing [46,47,48]. Additional supplementation with matrix vesicles to current treatments of calcium and vitamin D may be beneficial to mineralization, thus counteracting the softening of the bones [25]. Similarly, glycosaminoglycans with hyaluronic acid and its sulfated derivatives are capable of stimulating matrix vesicle-producing cells, thus increasing secretion of matrix vesicles [49]. This study found that aesculetin highly stimulated the formation of matrix vesicles harboring TNSALP and annexin V. Thus, one can assume that aesculetin may promote the formation of hydroxyapatite seed crystals in the sheltered interior of membrane-limited matrix vesicles bearing PHOSPHO1. Additionally, aesculetin enhanced the incorporation of osteoblasts into the mineralized collagen matrix with the help of thrombospondin-1 and tenascin C. However, this study did not examine the mechanisms that control the differentiation of osteoblasts into osteocytes embedded in bone collagenous matrix, which should be further investigated.

## 4. Materials and Methods

### 4.1. Materials

Minimum essential medium alpha medium (α-MEM), aesculetin and Alizarin red S dye were supplied by Sigma-Aldrich Chemical (St. Louis, MO, USA), as were all other reagents unless specifically stated otherwise. Fetal bovine serum (FBS), trypsin–EDTA, and penicillin-streptomycin were obtained from BioWhittaker (San Diego, CA, USA). 3-(4, 5-Dimetylthiazol-yl)-diphenyl tetrazolium bromide (MTT) was purchased from DUCHEFA Biochemie (Haarlem, The Netherlands). Antibodies of mouse collagen type 1 (cat. no. sc-293182, dilution 1:1000), mouse osteopontin (cat. no. sc-21742, dilution 1:2000), mouse osteocalcin (cat. no. sc-376835, dilution 1:500), mouse BSP II (cat. no. sc-73630, dilution 1:1000), mouse osteonectin (cat. no. sc-73472, dilution 1:1000), mouse annexin V (cat. no. sc-393669, dilution 1:1000), and mouse PHOSPHO1 (cat. no. sc-100351, dilution 1:1000), mouse thrombospondin-1 (cat. no. sc-12312, dilution 1:1000) and mouse TNSALP (cat. no. sc-137213, dilution 1:1000) were obtained from Santa Cruz Biotechnology (Dallas, TX, USA). Antibodies of rabbit OPG (cat. no. ab183910, dilution 1:1000) and mouse BMP-2 (cat. no. ab14933, dilution 1:1000) were provided by Abcam (Cambridge, UK). Rabbit tenascin C antibody (cat. no. 12221, dilution 1:1000) was provided from Cell Signaling (Danvers, MA, USA). Horseradish peroxidase (HRP)-conjugated goat anti-rabbit, goat anti-mouse and donkey anti-goat IgG were provided by Jackson Immuno Research Laboratories (West Grove, PA, USA).

Aesculetin was dissolved in dimethyl sulfoxide (DMSO) for live culture with cells; the final culture concentration of DMSO was <0.5%.

### 4.2. MC3T3-E1 Cell Culture and Osteoblastic Differentiation

MC3T3-E1 cell line (ATCC CRL-2593) was obtained from the American Type Culture Collection (Manassas, VA, USA) and cultured in α-MEM supplemented with 10% FBS, 100 U/mL penicillin and 100 μg/mL streptomycin at 37 °C with 5% CO_2_ in air. To differentiate MC3T3-E1 cells into osteoblasts, cells were seeded on 24-well plates at a density of 6.5 × 10^4^ cells, and cultured in α-MEM (differentiation media) supplemented with 10 mM β-glycerol phosphate, 50 μg/mL ascorbic acid and 100 nM dexamethasone for up to 21 days in the presence of 1–10 μM aesculetin. The media for cells were freshly replaced every 3 days.

Cytotoxicity of aesculetin was assessed by MTT assay, based on mitochondrial-dependent reduction of MTT to formazan crystal. MC3T3-E1 cells were seeded in 24-well plate at density 6.5 × 10^4^ cells and cultured in differentiation media for 3 or 21 days in the absence and presence of 1–20 μM aesculetin. After cell culture with aesculetin, 1 mg/mL MTT reagent was added to cells and incubated for 3 h at 37 °C with 5% CO_2_. Isopropanol was added to shed the formation of insoluble purple formazan product. Optical density was measured by using microplate reader at λ = 570 nm, corrected by reference wavelength at 690 nm.

### 4.3. Western Blot Analysis

MC3T3-E1 cells were seeded at six-well plates at a density 3 × 10^5^ cells/ml and cultured from day 3 to day 21 in differentiation media in the absence and presence of 1–10 μM aesculetin. Western blot analysis was conducted using cell lysates and supernatants prepared from cultured MC3T3-E1 cell-derived osteoblasts. Equal amounts of lysate proteins or equal volumes of culture media were electrophoresed on 6–20% SDS-PAGE gels and transferred onto a nitrocellulose membrane. Nonspecific binding was blocked by soaking membranes in a TBS-T buffer [50 mM Tris-HCl (pH 7.5), 150 mM NaCl, and 0.1% Tween 20] containing 3% bovine serum albumin or 5% nonfat milk for 3 h. The membranes were incubated with a primary antibody against collagen type 1, BMP-2, OPG, osteopontin, osteonectin, osteocalcin, BSP II, annexin V, PHOSPHO1, thrombospondin-1 or tenascin C. The membranes were then incubated with goat anti-rabbit, goat anti-mouse or donkey anti-goat IgG conjugated to HRP as a secondary antibody. The protein levels on gels were measured by using ECL chemiluminescent detection reagents (Millipore, Billerica, MA, USA) and Konica X-ray film (Konica, Tokyo, Japan). Incubation with β-actin antibody was conducted for comparative control.

### 4.4. Measurement of ALP Activity and ALP Staining

The ALP activity of MC3T3-E1 cells was performed on the day 7 during differentiation. Cells were lysed in 1.0% Triton X-100, followed by incubation with 0.5 M Tris–HCl (pH 9.9) containing 6 mM p-nitrophenyl phosphate (pNP) and 1 mM MgCl_2_ at 37 °C for 2 h. The protein contents were determined by Lowry assay, and the absorbance was read at λ = 405 nm in a microplate reader. The ALP activity was expressed as nmol pNP produced/min/mg protein.

The ALP staining was performed by using an ALP kit (Sigma-Aldrich Chemical). After the cells were cultured for seven days, cells were washed with phosphate buffered saline (PBS) and fixed with 4% formaldehyde, rinsed with 0.05% Tris-buffered saline-Tween 20 (mixture of Tris-buffered saline and 0.1% Tween 20), stained under protection from direct light. The ALP staining was conducted by adding naphthol/Fast Red Violet solution for 15 min as a substrate for cells. Naphthol/Fast Red Violet solution is a mixture of Fast Red Violet (0.8 g/L) with a 4 mg/mL Naphthol AS-BI phosphate solution in 2 M AMPD buffer (pH 9.5). Images for the visualization of ALP and its staining intensity were measured using an optical Axiomager microscope system (ECLIPSE TS100, Nikon, Tokyo, Japan).

### 4.5. Real-Time Polymerase Chain Reaction (PCR) Analysis

Following culture protocols, total RNA was isolated from MC3T3-E1 cells using a commercially available Trizol reagent kit. The level of mRNA transcripts of Runt-related transcription factor 2 (Runx2) was quantified by a 7500 real-time PCR system using Power SYBR Green PCR Master Mix (Thermo Fisher Scientific, Waltham, MA, USA). The primers (Bioneer, Daejeon, Korea) that were used to identify Runx2 gene were forward: 5′-AAGTGCGGTGCAAACTTTCT-3′, reverse: 5′-TCTCGGTGGCTGGTAGTGA-3′, 90 bp. Glyceraldehyde 3-phosphate dehydrogenase (GAPDH, forward primer: 5′-TTGTC AAGCTCATTTCCTGG-3′, reverse primer: 5′-GCCATGTAGGCCATGAGGTC-3′, 76 bp) was used as a reference gene to calculate the normalized expression of Runx2 gene. Quantitative PCR was carried out at 95 °C for 10 min, followed by 40 cycles of denaturation at 95 °C for 15 s and annealing at 60 °C for 60 s. Post-hold was performed at 4 °C.

### 4.6. Alizarin Red S Staining

For the measurement of calcium deposits, MC3T3-E1 cells were seeded on 24-well plate at density 6.5 × 10^4^ cells in differentiation media for 21 days in the absence and presence of 1–10 μM aesculetin. The medium culture was freshly changed every 3 days, and Alizarin red S staining was carried out on day 21. Cells were rinsed in cold PBS, fixed with 4% formaldehyde at room temperature for 15 min and stained with 40 mM Alizarin red S dye (pH 4.2) for 10 min. Calcium deposits were observed under light microscopy (ECLIPSE TS 100).

### 4.7. Immunofluorocytochemical Staining of TNSALP and Collagen Type 1

MC3T3-E1 cells were seeded on 24-well plate at density 6.5 × 10^4^ cells in differentiation media for 21 days in the absence and presence of 1–10 μM aesculetin. The medium culture was freshly changed every three days, and differentiated MC3T3-E1 cells were fixed with 4% formaldehyde for 10 min and permeated by 0.1% triton-x100 for 10 min on ice. To block the unspecific protein binding, differentiated cells were incubated with 20% FBS for 1 h. Subsequently, a primary antibody of TNSALP or collagen type 1 and a secondary antibody of fluorescein isothiocyanate (FITC)-conjugated or red Cy3-conjugated IgG were applied to cells. Nuclear counter-staining was carried out with 4′,6-diamidino-2-phenylindole (DAPI). Each slide was mounted in VectaMount mounting medium (Vector Laboratories, Burlingame, CA, USA). Images were taken using an optical Axiomager microscope system for the visualization of TNSALP (Zeiss, Oberkochen, Germany).

### 4.8. Data Analysis

The results are presented as mean ± SEM for each treatment group. Statistical analyses were performed using Statistical Analysis Systems statistical software package (SAS Institute Inc., Cary, NC, USA). Significance was determined by one-way ANOVA, followed by Duncan range test for multiple comparisons. Differences were considered significant at *p* < 0.05.

## 5. Conclusions

The current study demonstrated that aesculetin, a derivative of coumarin, enhanced osteoblastogenic differentiation and matrix vesicle-mediated collagen mineralization. Aesculetin stimulated the ALP activation and calcium deposit in MC3T3-E1 osteoblasts through well-functioning the BMP-2-Runx2 signaling. Concurrently, aesculetin boosted the induction of non-collagenous bone proteins of osteocalcin, osteonectin, osteopontin, and bone sialoprotein as well as collagen type 1 during de novo mineralization of osteoblasts. Furthermore, aesculetin accelerated release of matrix vesicles and adhesion of osteoblasts to preformed collagen fibrils, leading to deposition of hydroxyapatite crystals within bone collagenous matrix. Therefore, aesculetin can be a potent osteo-inductive compound boosting osteoblastogenesis and matrix vesicle-mediated mineralization.

## Data Availability

All of the data presented in this study are included in the article.

## Abbreviations

ALP: alkaline phosphatase; BMP-2, bone morphogenetic protein; BSP, bone sialoprotein; GSK-3β, glycogen synthase kinase-3β; MTT, 3-(4, 5-dimetylthiazol-yl)-diphenyl tetrazolium bromide; OPG, osteoprotegerin; Runx2, Runt-related transcription factor 2; TNSALP, tissue-nonspecific alkaline phosphatase.
